# Supplementary figures and images for: Experience-dependent serotonergic signaling in glia regulates targeted synapse elimination
Source: PLoS Biol. 2024 Oct 1;22(10):e3002822. doi: 10.1371/journal.pbio.3002822 (PMC11444420; doi:10.1371/journal.pbio.3002822)

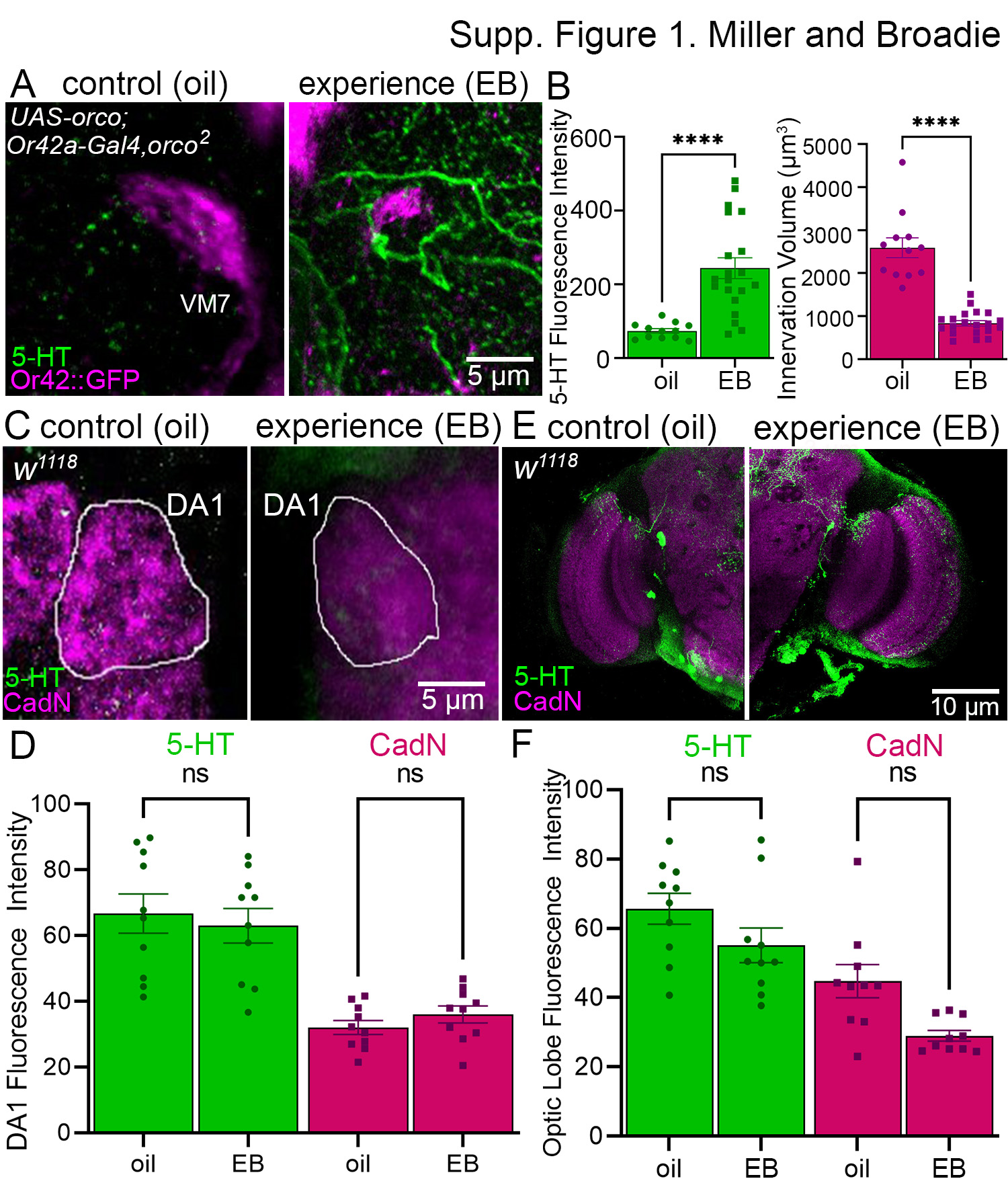

Supplement: S1 Fig — (A) UAS-orco driven with Or42a-Gal4 in orco null mutants (UAS-orco; Or42a-Gal4, orco2) enables the EB odorant response. Critical period exposure for 24 hours from 0–1 dpe to odorant oil vehicle (control, left) or 25% EB in oil (experience, right) with anti-serotonin (5-HT, green) and Or42a receptor-driven mCD8::GFP (Or42::GFP, magenta) membrane labeling for the VM7 innervation. (B) Quantification of 5-HT fluorescence intensity (left) and Or42a innervation volume (right) in VM7 glomeruli. Two-way ANOVA with Tukey’s multiple comparisons show a significant increase in serotonin (n = 12/condition, p = 7.13 × 10^−5^) and a significant decrease in VM7 innervation (n = 12/condition, p = 4.78 × 10^−10^) with EB experience. Individual data points with mean ± SEM. Significance indicated as p < 0.0001 (****). (C) Genetic background w1118 animals show no change in 5-HT (green) or CadN (magenta) in EB-independent DA1 glomeruli in response to 24-hour exposure from 0–1 dpe to odorant oil vehicle (control, left) or 25% EB in oil (experience, right). (D) Two-way ANOVA with Tukey’s multiple comparisons show no significant change in 5-HT (green, n = 10/condition, p = 0.930) or CadN (magenta, n = 10/condition, p = 0.0.910) fluorescence intensity with EB experience. (E) Genetic background w1118 animals show no change in 5-HT (green) or CadN (magenta) in the optic lobes in response to 24-hour exposure from 0–1 dpe to odorant oil vehicle (control, left) or 25% EB in oil (experience, right). (F) Two-way ANOVA with Tukey’s multiple comparisons show no significant changes in 5-HT (green, n = 10/condition, p = 0.2981) or CadN (magenta, n = 10/condition, p = 0.0535) fluorescence intensity with EB experience. Individual data points shown with mean ± SEM. Source data can be found in S1 Data. CadN, N-Cadherin; dpe, days post-eclosion; EB, ethyl butyrate; VM7, ventromedial 7. (TIFF) [file pbio.3002822.s001.tiff]

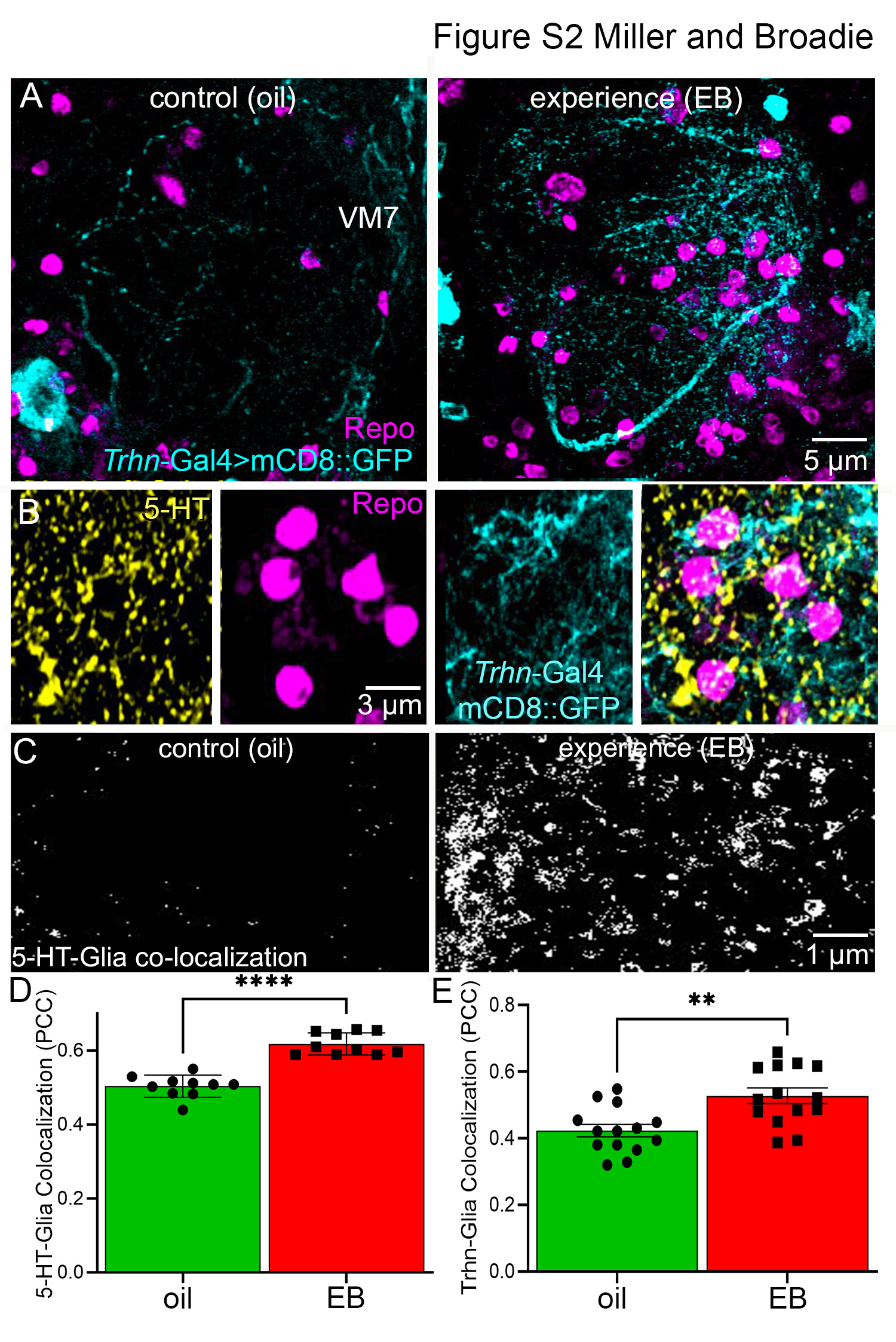

Supplement: S2 Fig — (A) Critical period (0–1 dpe) VM7 synaptic glomeruli colabeled for glial nuclei (Repo, magenta) and a Trhn-Gal4-driven UAS-mCD8::RFP membrane marker (cyan). The comparison shows 24-hour exposure from 0–1 dpe to the oil odorant vehicle control (left) and 25% EB (right). (B) High magnification imaging following EB exposure with triple-labeling for serotonin (5-HT, yellow), glial nuclei (Repo, magenta), and Trhn-Gal4-driven UAS-mCD8::RFP membrane marker (blue). (C) Colocalization of 5-HT and Repo labeling (white) following 24-hour exposure from 0–1 dpe to the oil odorant vehicle control (left) and 25% EB (right). (D) Quantification of 5-HT and glial Repo colocalization with the 2 treatment conditions. An unpaired t test comparison shows a significant increase in 5-HT-glia (Repo) colocalization (n = 10 each, p = 8.78 × 10^−8^) (E) Colocalization quantification of glial Repo and the Trhn-Gal4-driven UAS-mCD8::RFP membrane marker with the 2 treatment conditions. An unpaired t test comparison shows significant increase in Trhn-glia (Repo) colocalization (n = 10 each, p = 0.0017). Source data can be found in S1 Data. dpe, days post-eclosion; EB, ethyl butyrate; Trhn, tryptophan hydroxylase; VM7, ventromedial 7. (TIFF) [file pbio.3002822.s002.tiff]

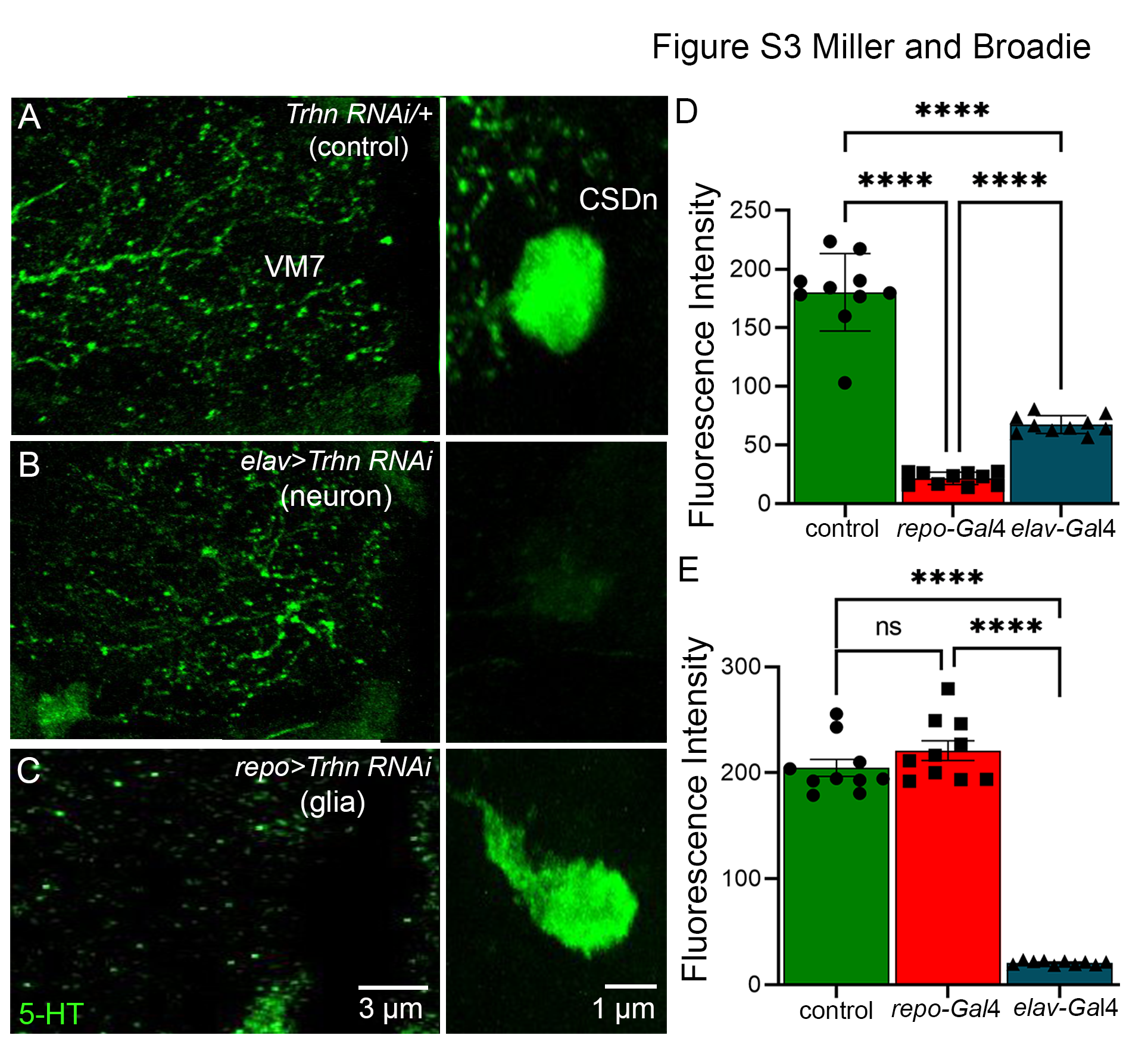

Supplement: S3 Fig — (A) UAS-Trhn RNAi/+ transgenic control with critical period exposure for 24 hours from 0–1 dpe to 25% EB experience, with serotonin labeling (5-HT, green) in the VM7 glomerulus (left), and serotonergic neuron (CSDn) cell body (right). (B) Trhn RNAi driven by elav-Gal4 in neurons (elav>Trhn RNAi) under identical conditions. The EB experience-dependent serotonin up-regulation persists in the VM7 glomerulus (left), although serotonin is lost in the CSDn soma as expected (right). (C) Trhn RNAi driven by repo-Gal4 in glia (repo>Trhn RNAi) under identical conditions. The experience-dependent serotonin up-regulation is lost in the VM7 glomerulus (left), although serotonin is maintained within the CSDn soma (right). (D) Quantification of 5-HT fluorescence intensity in VM7. One-way ANOVA with Tukey’s multiple comparison shows significant decrease in 5-HT in the repo-Gal4-driven UAS-Trhn RNAi compared to the UAS-Trhn RNAi/+ control (n = 10, p = 9.00 × 10^−15^) and elav-Gal4-driven UAS-Trhn RNAi (n = 10, p = 5.72 × 10^−5^). (E) Quantification of 5-HT fluorescence intensity in the CSDn neuronal cell body. One-way ANOVA with Tukey’s multiple comparison shows a significant decrease in 5-HT in elav-Gal4 compared to repo-Gal4-driven UAS-Trhn RNAi (n = 10, p = 8.00 × 10^−15^). All individual data points are shown with the mean ± SEM. Significance is indicated as p < 0.0001 (****), p < 0.05 (*), and p > 0.05 (not significant, ns). Source data can be found in S1 Data. CSDn, contralaterally projecting, serotonin-immunoreactive deutocerebral neurons; dpe, days post-eclosion; EB, ethyl butyrate; RNAi, RNA interference; Trhn, tryptophan hydroxylase; VM7, ventromedial 7. (TIFF) [file pbio.3002822.s003.tiff]

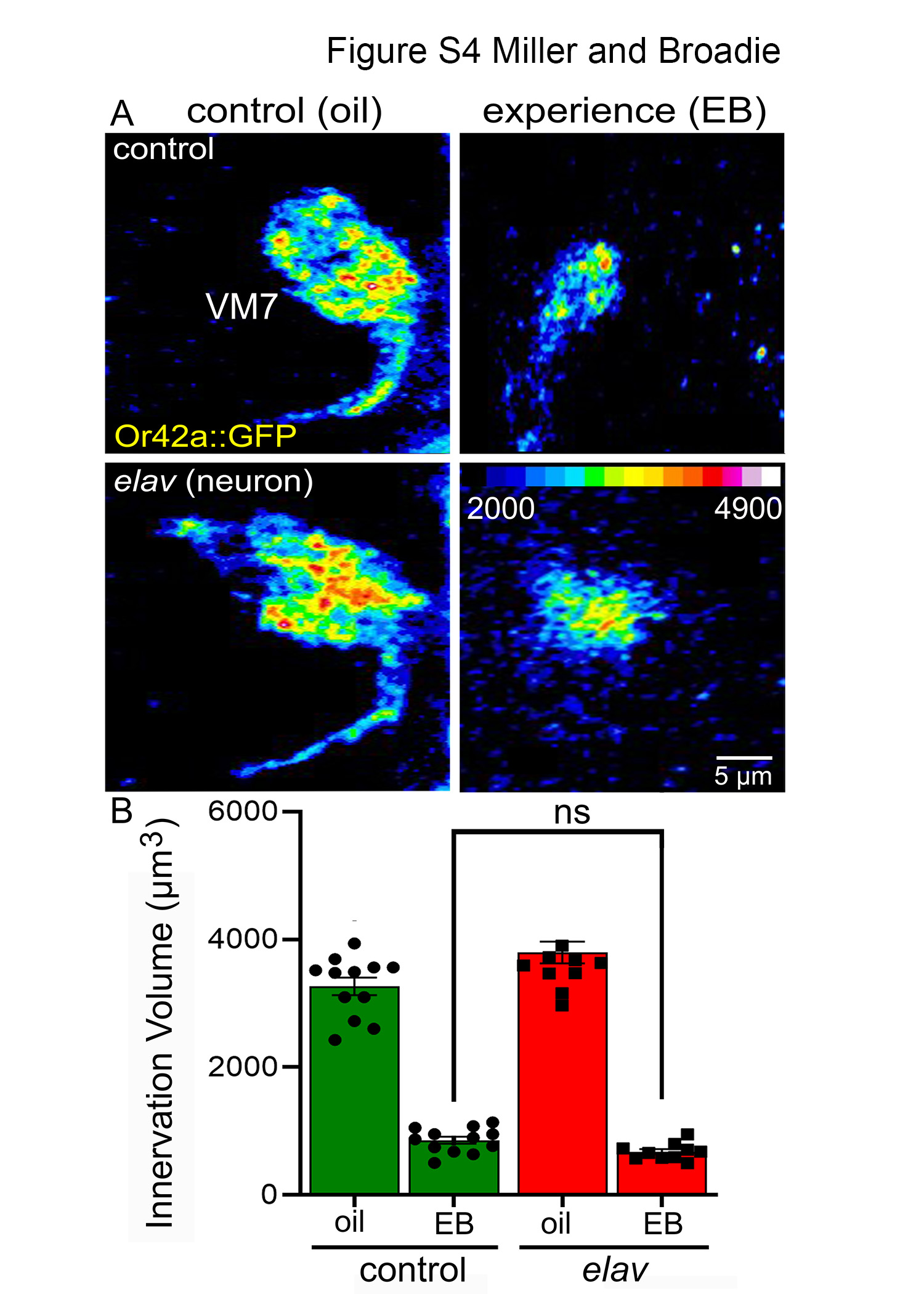

Supplement: S4 Fig — (A) 5-HT2AR RNAi control (w1118; Or42a-mCD8::GFP/+; UAS-5-HT2AR RNAi/+; top) and neuron-specific 5-HT2AR RNAi (w1118; Or42a-mCD8::GFP/elav-Gal4; UAS-5-HT2A RNAi/+, bottom) following critical period exposure for 24 hours from 0–1 dpe to odorant oil vehicle (control, left) or 25% EB (experience, right). The Or42a-mCD8::GFP innervation of the VM7 glomerulus shown as a heat-map based on intensity (color LU scale; lower right panel). (B) Quantification of the Or42a neuron innervation 3-D volume. Two-way ANOVA with Tukey’s multiple comparison shows significant pruning with EB experience (n = 12/condition, p = 1.05 × 10^−12^), which is not significantly different from the neuron-targeted 5-HT2AR RNAi condition (n = 10/condition, p = 0.73). Individual data points are shown with mean ± SEM. Significance indicated as p > 0.05 (not significant, ns). Source data can be found in S1 Data. dpe, days post-eclosion; EB, ethyl butyrate; RNAi, RNA interference; VM7, ventromedial 7. (TIFF) [file pbio.3002822.s004.tiff]

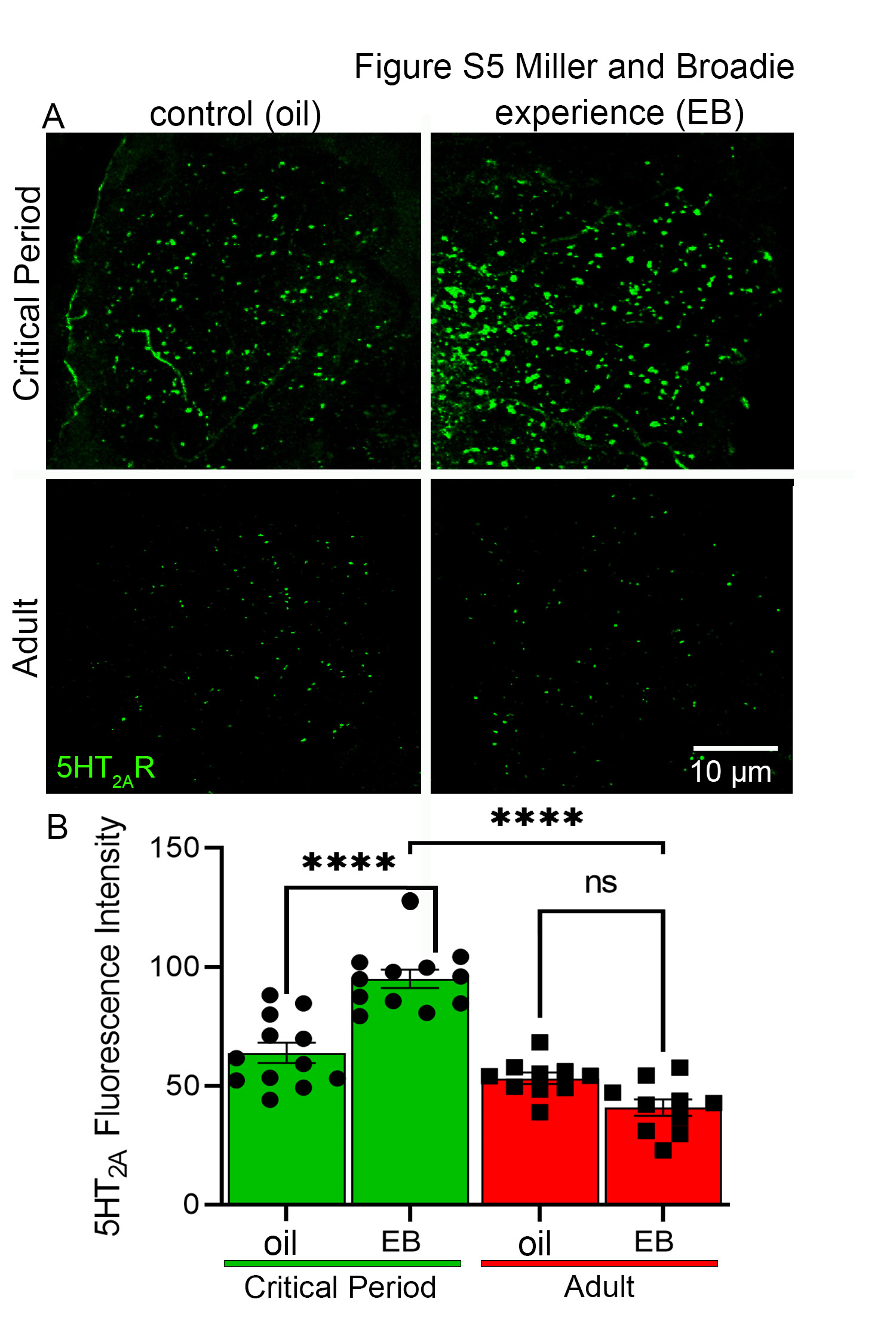

Supplement: S5 Fig — (A) Control (w1118) juvenile critical period (0–1 dpe, top) or mature adult (7–8 dpe, bottom) AL staining of the 5HT2A receptors (anti-5HT2AR, green) following exposure for 24 hours to oil (control, left) and 25% EB in oil (experience (EB), right). (B) Quantification of 5HT2AR fluorescence intensity in both time periods and treatment conditions. Two-way ANOVA with Tukey’s multiple comparison shows significant up-regulation in 5HT2AR fluorescence intensity within the juvenile critical period with EB experience (green, left, n = 10/condition, p = 1.12 × 10^−6^), but no significant change in mature adults (red, right, n = 10/condition, p = 0.123). All individual data points are shown with mean ± SEM. Significance indicated as not significant (ns) at p > 0.05. Source data can be found in S1 Data. AL, antennal lobe; EB, ethyl butyrate; 5-HT2AR, 5-HT2A receptor. (TIFF) [file pbio.3002822.s005.tiff]

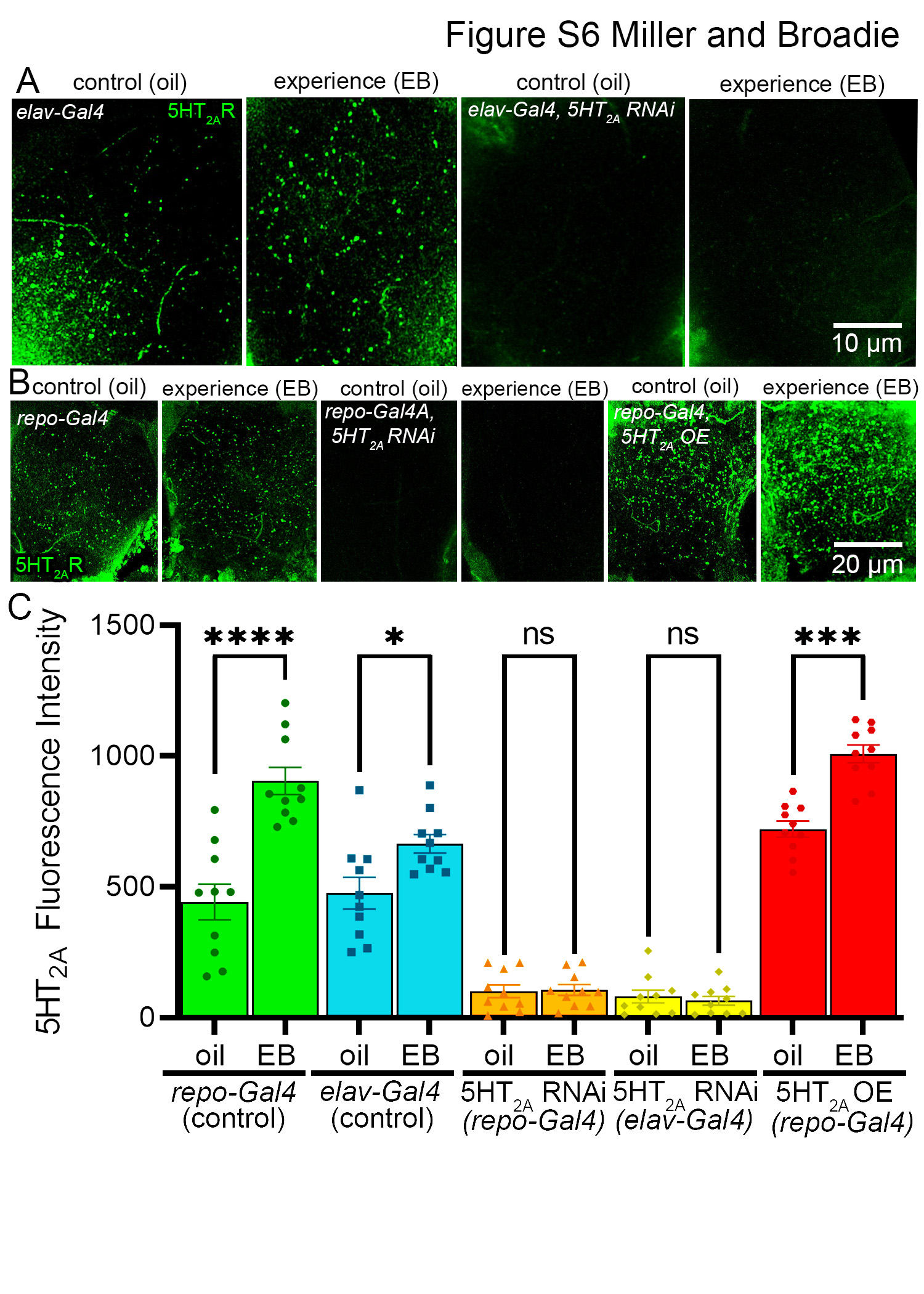

Supplement: S6 Fig — (A) Neuronal transgenic control (elav-Gal4/+, left) and neuronal knockdown of 5HT2A receptors (elav-Gal4/+, UAS-5HT2A RNAi/+, right) with 5HT2A receptor labeling (green) following critical period exposure for 24 hours from 0–1 dpe to odorant vehicle (control, oil) or 25% odorant (experience, EB). (B) The same labeling with glial repo-Gal4 control (repo-Gal4/+, left), glial knockdown of 5HT2A receptors (UAS-5-HT2A RNAi/ repo-Gal4, middle), and glial 5HT2A OE (UAS-5-HT2A OE/ repo-Gal4, right). (C) Quantification of 5HT2AR fluorescence intensity in the repo-Gal4 control (green), elav-Gal4 control (blue), repo-Gal4 5HT2A RNAi (orange), elav-Gal4 5HT2A RNAi (yellow, second from the right), and 5HT glial 5HT2A OE (red). Two-way ANOVA with Tukey’s multiple comparison shows EB experience-dependent increase in 5HT2A receptor levels in both transgenic controls (n = 10 each, repo-Gal4; p = 5.38 × 10^−10^, elav-Gal4; p = 0.0205), a significant decrease in 5HT2A receptor levels in both RNAi conditions (n = 10 each, repo-Gal4; p = 4.63 × 10^−10^, elav-Gal4; p = 1.05 × 10^−9^), and a significant elevated EB response with glial 5HT2AR OE (n = 10/condition, p = 2.36 × 10^−9^). All individual data points are shown with the mean ± SEM. Significance is indicated as p < 0.0001 (****), p < 0.05 (*), and p > 0.05 (not significant, ns). Source data can be found in S1 Data. dpe, days post-eclosion; EB, ethyl butyrate; OE, overexpression; RNAi, RNA interference. (TIFF) [file pbio.3002822.s006.tiff]

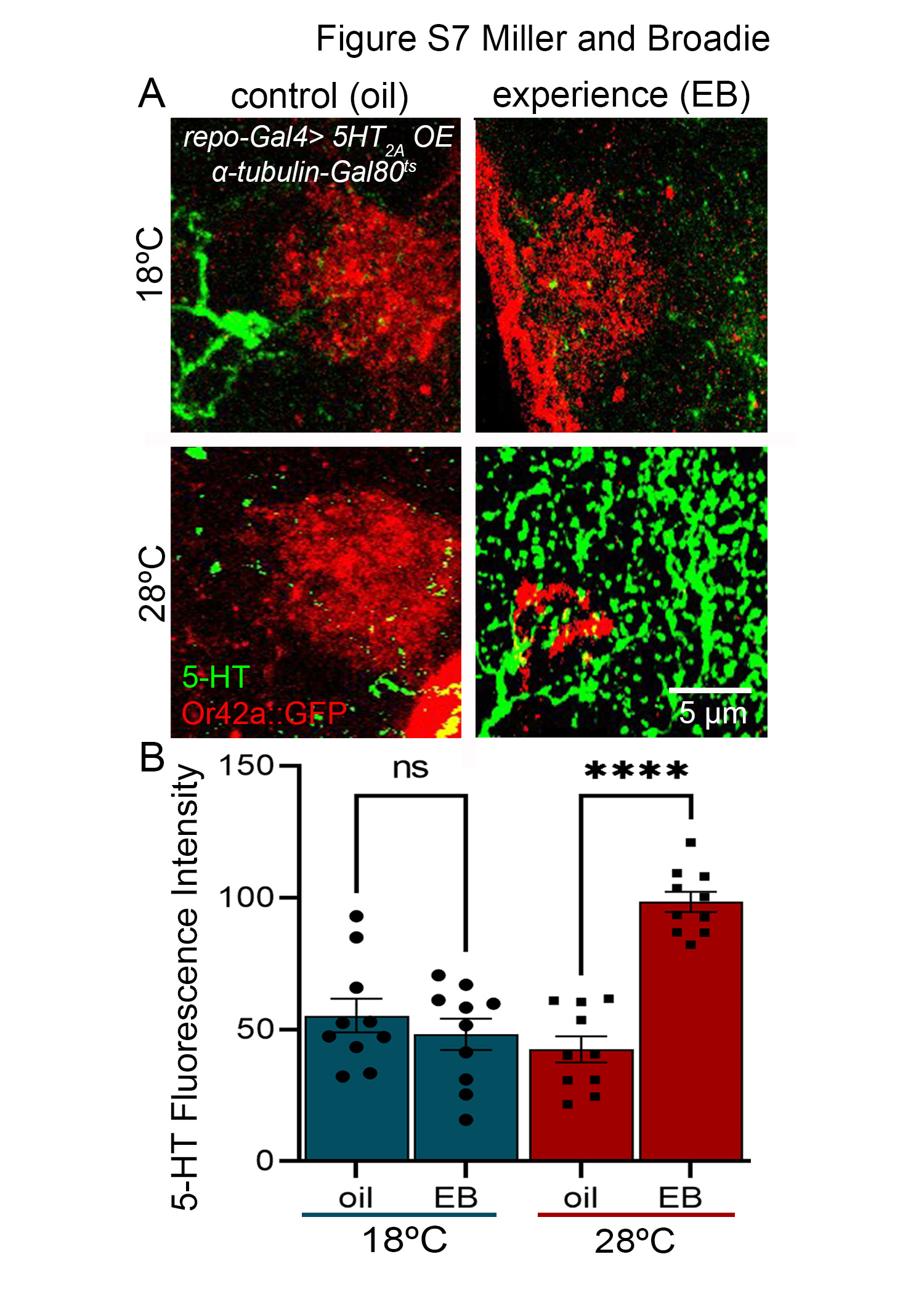

Supplement: S7 Fig — (A) The conditional glial 5HT2AR OE line (w1118; tubulin-Gal80ts/Or42a::GFP; UAS-5HT2AR OE/ repo-Gal4) in the permissive 18°C Gal80ts-repressed condition (top) and restrictive 28°C 5HT2AR OE condition (bottom). VM7 colabeling for serotonin (5-HT, green) and Or42a innervation (Or42a::GFP, red) following mature adult exposure for 24 hours to either odorant vehicle (oil, left) or 25% odorant (EB, right). (B) Quantification of 5-HT fluorescence intensity in the permissive 18°C Gal80ts-repressed (blue) and restrictive 28°C 5-HT2AR OE (red) conditions, in the oil control and EB exposure. Two-way ANOVA with Tukey’s multiple comparison tests show no significant change in 5-HT fluorescence intensity in the 18°C control (n = 10 each, p = 0.789), but a significant increase with glial-targeted 5HT2AR OE at 28°C (n = 10 each, p = 6.81 × 10^−8^). Individual data points are shown with mean ± SEM. Significance is indicated as p < 0.0001 (****) and p > 0.05 (not significant, ns). Source data can be found in S1 Data. EB, ethyl butyrate; OE, overexpression; VM7, ventromedial 7. (TIFF) [file pbio.3002822.s007.tiff]

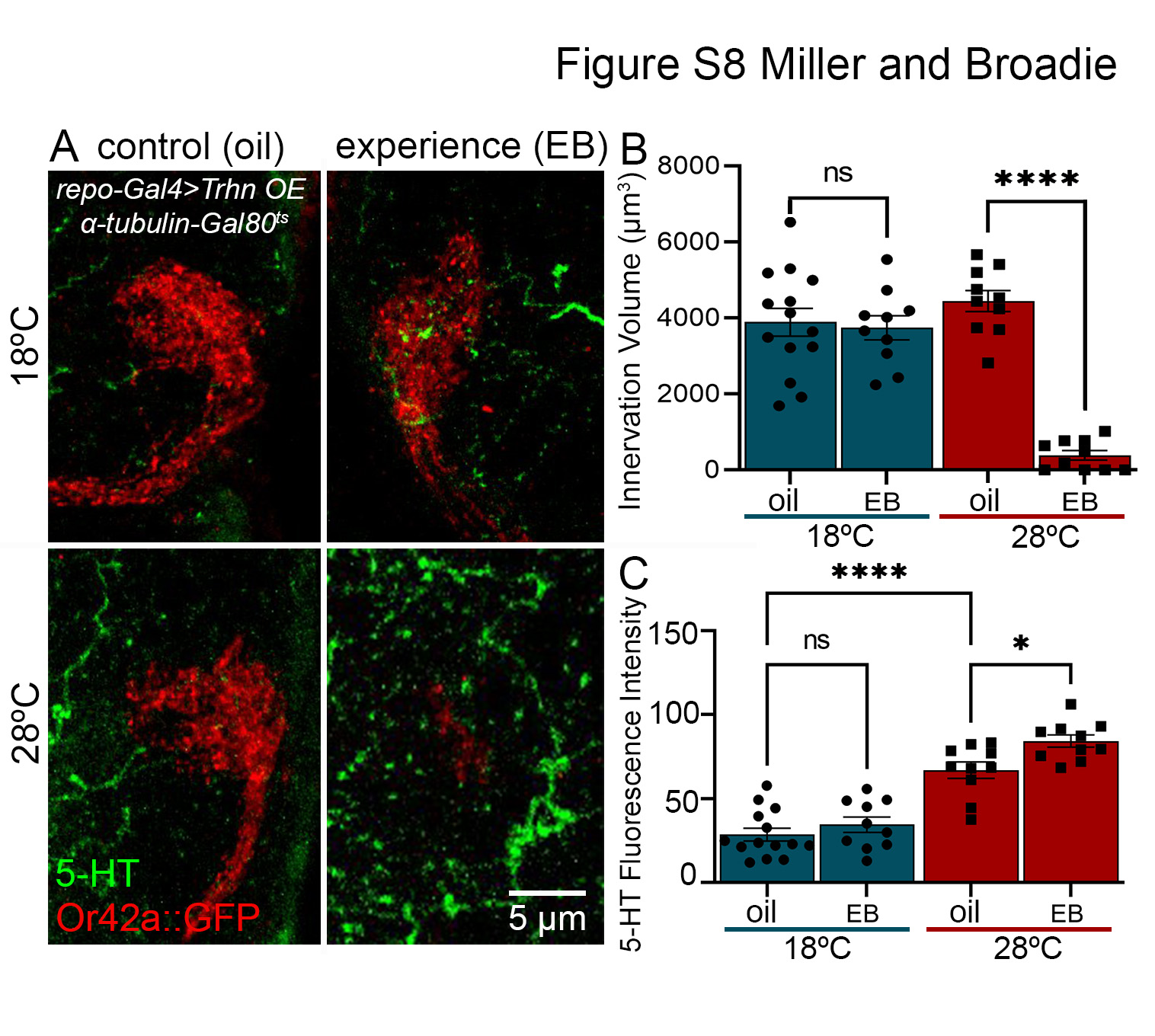

Supplement: S8 Fig — (A) The conditional glial Trhn OE line (w1118; tubulin-Gal80ts/UAS-Trhn OE; repo-Gal4/UAS-Or42a::GFP) in the permissive 18°C Gal80ts-repressed condition (top) and 28°C Trhn glial OE condition (bottom). VM7 colabeling for serotonin (5-HT, green) and Or42a innervation (Or42a::GFP, red) following mature adult exposure for 24 hours to either odorant vehicle (oil, left) or 25% odorant (EB, right). (B) Quantification of synaptic glomeruli innervation volumes in 18°C control (blue) and 28°C Trhn OE (red) conditions. Two-way ANOVA with Tukey’s multiple comparison tests show no significant change in innervation volume at 18°C (n = 10 each; p = 0.985), but a significant decrease at 28°C with glial Trhn OE (n = 10 each, p = 3.53 × 10^−10^). (C) Quantification of 5-HT fluorescence intensities in 18°C control (blue) and 28°C Trhn OE (red) conditions. Two-way ANOVA with Tukey’s multiple comparison tests show no significant change in 5-HT at 18°C (n = 10 each, p = 0.740), but a significant increase at 28°C with glial Trhn OE (n = 10 each, p = 0.0412). Individual data points are shown with mean ± SEM. Significance is indicated as p < 0.0001 (****), p < 0.05 (*), and p > 0.05 (not significant, ns). Source data can be found in S1 Data. EB, ethyl butyrate; OE, overexpression; Trhn, tryptophan hydroxylase; VM7, ventromedial 7. (TIFF) [file pbio.3002822.s008.tiff]
