# Supplementary material for: Experience-dependent serotonergic signaling in glia regulates targeted synapse elimination
Source: PLoS Biol. 2024 Oct 1;22(10):e3002822. doi: 10.1371/journal.pbio.3002822 (PMC11444420; doi:10.1371/journal.pbio.3002822)
Supplement: Attachment — Submitted filename: Response to Reviwers_Miller and Brodie 2024.docx [file pbio.3002822.s010.docx]

Dear Editor,

We submit here our invited revision within your specified submission deadline. Our point-by-point responses to all 3 reviewer suggestions are given below. This revision has 6 new figures; 2 main and 4 supplemental figures. All other text and figure revisions are listed below in the reviewer responses. We have done our best to comply with all final submission guidelines. However, if you need any further paper materials whatsoever, please just let us know and we will respond quickly.

**Review Response:** Experience-Dependent Glial Serotonin Self-Signaling Sculpts Brain Circuitry

Vanessa Miller and Kendal Broadie

Reviewer 1

This study seeks to determine if serotonin signaling impacts a critical period in the olfactory system of Drosophila that depends upon glial cells. This group recently demonstrated that glia are responsible for a reduction in glomerular volume that is triggered by exposure to a high concentration of the cognate odorant for that glomerulus. In this study, they build upon this work to study the role of serotonin released by glia to act upon a glia expressed serotonin receptor. Unfortunately, there are several aspects of the data provided that raise concern which include quality of images and too little validation demonstrating that the genes targeted by RNAi are expressed by glia.

Major concerns:

- The claim that glial cells synthesize and release serotonin in the brain of Drosophila is a very novel and requires strong supportive evidence, however very little evidence is provided. There are low-resolution immunolabeling images in which there may be colocalization between Repo-Gal4 (supplementary figure 2) and serotonin labeling, but they are not convincing because they are so cropped that they lack perspective and do not show glial cell bodies at all (supplementary figure 3). The other evidence is indirect in nature in which a tryptophan hydroxylase RNAi construct expressed in a glial driver line (Repo-Gal4) prevents the plasticity from occurring. There is no direct evidence demonstrating that glial cells synthesize serotonin, such as serotonin labeling of glial cell bodies or fluorescent in situ hybridization or transcriptomic analysis showing expression of serotonin synthesis enzymes. Simply checking FlyCell Atlas (a free online single-cell RNAseq dataset), there is no evidence for co-expression of repo with tryptophan hydroxylase or aromatic amino acid decarboxylase. Some form of direct evidence that glia produce serotonin is needed.

As suggested, we have added new experiments of a tryptophan hydroxylase (Trhn)-Gal4 driven membrane marker (UAS-mCD8::GFP), serotonin (anti-5-HT), and a glial cell body nuclear marker (anti-Repo) to further demonstrate serotonin synthesis in glia (new Figure S2). We also do new studies with critical period experience (Fig. S2A-C) to show increased co-localization of both the Trhn marker and 5-HT in the glial cell bodies (Fig. S2D,E). Combined with the other data outlined by the reviewer, and shown in the paper, this provides direct evidence that glia produce serotonin.

- The justification for testing the role of the 5-HT2A receptor in glial pruning is that this serotonin receptor is expressed by mammalian glia. However, the 5-HT2A receptor in Drosophila is not a homolog of the mammalian 5-HT2A nor are the glia cells necessarily of similar cell types across these disparate groups. There needs to be some evidence that glia in Drosophila express the 5-HT2A receptor, otherwise expressing an RNAi construct with no validation of efficacy or off-target effects is not sufficiently rigorous to make any conclusion. Similar to the point above, a quick check in FlyCell atlas shows no expression of the 5-HT2A by glia in the brain of Drosophila, so there is a risk that effects caused by expressing could be artifactual without direct evidence that the 5-HT2A receptor is expressed by glia.

To our best knowledge, *Drosophila* 5-HT_2A_R is a homolog for the mammalian 5-HT_2A_ receptor. NCBI and OrthoDB both indicate this homology. This link shows 5-HT_2A_R alignment for *Drosophila* vs. human: [*https://www.flyrnai.org/cgi-bin/DRSC_prot_align.pl?geneid1=40575&geneid2=3356*](https://www.flyrnai.org/cgi-bin/DRSC_prot_align.pl?geneid1=40575&geneid2=3356)

*Drosophila* glia have been shown to have conserved functions, including our demonstrations of experience-dependent synapse pruning during critical periods. Although glia naming conventions are quite different, *Drosophila* glia act as similar cell types compared to the glia in other species. As suggested for 5-HT_2A_R, we pursued major experiments with new 5-HT_2A_R antibody labeling (new Figures S5 and S6). Consistent with the homology above, the mammalian 5-HT_2A_R antibody recognizes *Drosophila* 5-HT_2A_R. To validate this antibody, we first drove 5-HT_2A_R RNAi in neurons with *elav*-Gal4 to show a loss of the receptors (Fig. S6A,C). In glia, we drove both 5-HT_2A_R RNAi and 5-HT_2A_R overexpression with *repo*-Gal4 to show a loss of glia-specific 5-HT_2A_R with RNAi and elevation of 5-HT_2A_R with glial overexpression (Fig. S6B,C). These extensive revision data provide new direct evidence that the 5-HT_2A_ receptor is expressed by glia, and validate our tools.

- The increase in serotonin immunolabeling during the ethyl butyrate treatment as shown in figure 1C has two major flaws. First, the pattern of increased expression is clearly neuronal in nature with obvious branching compartments (Figure 1C and supplementary figure 1A) that are not consistent with reports of glial structure. This contradicts the suggestion that glial pruning is due to changes in glial, rather than neuronal, production of serotonin. The second issue is that the representative images are heavily cropped without showing serotonin labeling in the remainder of the brain. If this is a process involving the olfactory system, then there should be no change in labeling intensity in other brain regions such as the optic lobes. Fluorescence intensity measures must have internal controls (i.e. measures of fluorescence intensity from sources that are not impacted by a given manipulation) to ensure that any changes in fluorescence are not due to differences in confocal settings or other factors.

The 5-HT labeling includes both neurons and glia. We have recently published that glia infiltrate the synaptic neuropil during the experience-dependent critical period via branched projections. Moreover, we show glial-specific tryptophan hydroxylase knockdown results in a most significant reduction in 5-HT fluorescence intensity within the antennal lobe synaptic neuropil (new Fig. S3D), whereas the 5-HT fluorescence intensity remains unaltered in the CSDn serotonergic neurons (new Fig. S3E). Thus, increased 5-HT glomerular levels are driven by glial serotonin production. As suggested, we have assayed serotonin labeling in other brain regions compared to an internal labeling control (revised Figure S1). We find there is no significant odorant experience-dependent upregulation of 5-HT in the EB non-responsive DA1 glomerulus (Fig. S1C,D), or in the optic lobes (Fig. S1E,F). As the fluorescence intensity internal control, these experiments are compared with N-Cadherin, which is likewise not significantly affected by ethyl butyrate olfactory experience. Thus, experience-dependent serotonin upregulation is both glia-specific and brain region-specific.

Reviewer 2

In this manuscript, Miller and Broadie report that autocrine serotonin signaling in glia regulates olfactory experience-dependent pruning in juvenile brains. Based on their studies, they also propose that expression of serotonin 5-HT2A autoreceptors is sufficient to re-open the critical developmental plasticity period in mature adults. This leads them to propose that glial serotonin autocrine signaling may yield intervention strategizes to alter experience-dependent plasticity remodeling in disease state. This study advances the field in two ways. One, it reveals a role for glial autocrine serotonergic signaling in pruning. Two, it suggests how glial pruning is limited to developmental critical periods. This is a significant contribution that will be of interest to the fields of glia biology, developmental neurobiology, and neurological disease modeling. To make this work suitable for publication in PLOS Biology, the following points should be addressed:

Major Comments:

1. To support their conclusions, the authors need to demonstrate that EB does not change 5HT levels in non-VM7/Or42a innervated glomeruli, in at least one condition (wild type/Fig 1 or Or42a restricted/Fig S1).

As suggested, we have assayed serotonin labeling in a non-VM7/Or42a innervated glomerulus (revised Figure S1). We find there is no detectable EB odorant experience-dependent change of serotonin (5-HT) levels within the EB non-responsive DA1 synaptic glomerulus (new Fig. S1C,D).

2. It is unclear how over-expression of the receptor alone is sufficient to prune in mature adults. The authors should show 5HT ligand staining with receptor OE and/or if TrpH over-expression phenocopies this adult reopening of plasticity.

As suggested, we have tested serotonin labeling with glia 5-HT_2A_R overexpression (new Fig. S7). In this condition, we find that 5-HT is upregulated in mature adults consistent with induced pruning. Moreover, we find TrpH overexpression phenocopies 5-HT_2A_R overexpression (new Fig. S8).

3. The authors should show whether 5HT2AR levels change between juveniles (critical period) and mature adults to explain the critical period window per their model.

As suggested, we have compared 5HT_2A_R levels in juveniles and adults (new Fig. S5). We find EB experience-dependent upregulation of 5HT_2A_R is restricted to the critical period. This suggests elevated levels of the 5HT_2A_ receptor during the critical period is important for defining the window.

4. Given prior implication of Draper in VM7 plasticity and altered expression with age, authors should examine Draper levels in juvenile EB and 5HT2AR OE conditions.

We have not been able to achieve reliable Draper labeling in the *Drosophila* brain (see our Nature Comm. 12(1):1160, 2021), and therefore have not been able to pursue this excellent suggestion.

Minor Comments:

5. Some relevant references are missing and should be added e.g. PMID: 24270812, PMID: 33828296, PMID: 24361692, PMID: 37333889, PMID: 30150774, PMID: 25857335, PMID: 33156956

These references have been added to the relevant locations in the revised manuscript.

6. Line 42-44: The distinction made between "elimination…during experience dependent pruning" and "mediating pruning during experience dependent critical period" is unclear. Rephrase suggested.

This distinction has been clarified.

7. A schematic in Figure 1 of the glomerulus and plasticity will be helpful to orient the reader.

Figure 1 is too large to add more, so we have added a new orientation figure (new Figure 1).

8. Image quality needs improving. Some images/labels are hard to discern, for e.g.: -Fig 1A bottom right: is the text REPO? RFP? What is shown - glial projections are not visible or labeled by arrows. In Fig 1A top left, is the glomerulus outside the antennal lobe? -Fig 1C will be helped by also showing individual color panels. -Fig S2A top panels: what is to be inferred? There seem more glia cells in Fig S2A vs B, which makes the 5HT staining hard to infer. Schematic of which glia imaged will help.-Fig 2B: red and orange (repo vs elaV) colors are nearly identical is suggested that each panel is labeled for easy referencing for the reader.

This figure labeling is clarified. We have black-highlighted the REPO::RFP label in Fig. 1A. Glial projections are not visible. The glomerulus is within the antennal lobe, but labeled in the other brain hemisphere for clarity. We have added further examples of Fig. 1C labeling in the many new supplementary figures. We have added an entirely new figure to address the glial questions (new Figure S2). We have added a new schematic to illustrate the experience-dependent infiltrating glial projections (new Figure 6). The graph labels in original Fig. 2B clearly distinguish genotypes.

9. It is suggested that each panel is labeled for easy referencing for the reader.

All panels that are independently cited in the text have been labeled.

10. Overall, the text requires editing throughout for clarity and conciseness. For example, Line 148-149 is redundant with Line 159-160, since Fig 2B is a quantification of Fig. 2A.

We have worked to improve the clarity and conciseness of the revised manuscript.

11. Repo:GAL4 expresses in multiple glia. The authors should clarify in text if they consider different glia to be signaling/receiving units or truly autocrine at single-cell level.

We have clarified this point in the revised manuscript.

12. Line 161 needs statistical comparison in corresponding data in Fig 2B.

 A statistical comparison has been added to the revised manuscript.

Reviewer 3

Major points:

1) The fact that neurons are not involved in this experience-dependent synaptic pruning is based only upon lack of effects of RNAi constructs of both Trhn-RNAi and 5-HT2A-RNAi (Figure 2 and Sup. Figure 3). It must be shown that these RNAi are both indeed working in the neurons by clearly reducing the level, obtained by immunolabeling or qRT-PCR, of 5-HT and receptor.

As suggested, we validated both Trhn and 5-HT_2A_R RNAi in neurons (new Figures S3 and S6). Neuronal Trhn RNAi causes an overall decrease in 5-HT levels and a specific loss in the CSDn neurons; the serotonergic neurons innervating the antennal lobe (Fig. S3B, D, and E). Likewise, the neuronally-targeted 5-HT_2A_R RNAi causes the loss of 5HT_2A_ receptor expression (Fig S6A,C).

2) The experience showing the re-open experience dependent synaptic pruning by adult receptors (Figure 4) should be bolstered by showing that this effect is indeed due to the concomitant upregulation of 5-HT. Also, the effect due to the glial expression of UAS-5-HT2A described should be abolished if UAS-Trhn-RNAi is co-expressed at the same time compared to a neutral UAS to have the same number of UAS present.

As suggested, we now demonstrate the dual effects of glial 5HT_2A_R overexpression induction of 5-HT signaling and direct Trhn overexpression in mature adult glia for *de novo* synaptic pruning (new Figures S7 and S8). We also tried to generate animals for dual glial expression of 5HT_2A_R and Trhn RNAi, but have not been able to produce viable animals with this crossing combination.

3) The authors claim an autocrine mechanism, but this is not proven since they are using only repo-GAL4 which is expressed in all glia cell types. 5-HT could well be produced by one type of glia and used by another type that expressed the 5-HT2A receptor. Either the authors add results with a specific type of glia which show pruning defects with both Trhn-RNAi and 5-HT2A-RNAi, or they must remove the autocrine mechanism as proven.

As suggested, we have removed the autocrine mechanism as proven and re-worded the revision.

4) Why Figure S2 and S3 are not quantified? These quantifications must be done.

As suggested, the full quantifications have been added to both original Figures S2 and S3.

Minor points:

1) It will be very helpful for the readers to have a schematic model of the role of glia in this experience-dependent pruning.

As suggested, a schematic model figure has been added (new Figure 6).

2) There is no mention of early work on Drosophila where glia cells were first recognized as phagocytes in axon pruning during mushroom body neuronal remodeling (Awasaki et al., 2004 and Watts et al., 2004). This is even more surprising that the corresponding author wrote a dispatch in the same issue of Current Biology to highlight these two seminal papers.

These references have been added to the revised manuscript.

3) line 367: Bloomington stock (25842) official name is Trhn RNAi and not TRH RNAi. This must be changed in all the manuscript, figures included.

This change has been made throughout the revised manuscript.

4) serotoninergic appears several times in the manuscript instead of serotonergic.

These typos have been corrected in the revised manuscript. Also, please note that there is no minor point 5 in this review; numbers skip from 4 to 6. We keep to this numbering scheme here.

6) Specify the complete genotype used in Figure 1 and Figure S2 figure legends.

The full genotypes have been added to Figure 1 and S2 figure legends.

7) It will be helpful to add UAS-Trhn RNAi in all appropriate panels in Figure 2.

Trhn RNAi labels have been added to original Figure 2.

8) The genotypes are unclear in the Figure 4 panels.

The genotypes have been added to the Figure 4 panels.
